# ANDC: an early warning score to predict mortality risk for patients with Coronavirus Disease 2019

**DOI:** 10.1186/s12967-020-02505-7

**Published:** 2020-08-31

**Authors:** Zhihong Weng, Qiaosen Chen, Sumeng Li, Huadong Li, Qian Zhang, Sihong Lu, Li Wu, Leiqun Xiong, Bobin Mi, Di Liu, Mengji Lu, Dongliang Yang, Hongbo Jiang, Shaoping Zheng, Xin Zheng

**Affiliations:** 1 Department of Infectious Diseases, Union Hospital, Tongji Medical College, Huazhong University of Science and Technology, 1277 JieFang Avenue, Wuhan, 430022 China; 2 Joint International Laboratory of Infection and Immunity, Union Hospital, Huazhong University of Science and Technology, Wuhan, China; 3 Department of Epidemiology and Biostatistics, School of Public Health, Guangdong Pharmaceutical University, 283 Jianghai Road, Guangzhou, 510310 China; 4 Department of Infectious Diseases, Wuhan Jinyintan Hospital, Wuhan, China; 5 Department of Gastroenterology, Loudi Central Hospital, Loudi, China; 6 Department of Tuberculosis, Wuhan Pulmonary Hospital, Wuhan, China; 7 Department of Orthopedics, Union Hospital, Tongji Medical College, Huazhong University of Science and Technology, Wuhan, China; 8 Pritzker School of Medicine, University of Chicago, Chicago, USA; 9 Institute of Virology, University Hospital Essen, Essen, Germany; 10 Department of Ultrasound, Union Hospital, Tongji Medical College, Huazhong University of Science and Technology, 1277 JieFang Avenue, Wuhan, 430022 China

**Keywords:** SARS-Cov-2, COVID-19, Nomogram, Mortality, Risk factor

## Abstract

**Background:**

Patients with severe Coronavirus Disease 2019 (COVID-19) will progress rapidly to acute respiratory failure or death. We aimed to develop a quantitative tool for early predicting mortality risk of patients with COVID-19.

**Methods:**

301 patients with confirmed COVID-19 admitted to Main District and Tumor Center of the Union Hospital of Huazhong University of Science and Technology (Wuhan, China) between January 1, 2020 to February 15, 2020 were enrolled in this retrospective two-centers study. Data on patient demographic characteristics, laboratory findings and clinical outcomes was analyzed. A nomogram was constructed to predict the death probability of COVID-19 patients.

**Results:**

Age, neutrophil-to-lymphocyte ratio, d-dimer and C-reactive protein obtained on admission were identified as predictors of mortality for COVID-19 patients by LASSO. The nomogram demonstrated good calibration and discrimination with the area under the curve (AUC) of 0.921 and 0.975 for the derivation and validation cohort, respectively. An integrated score (named ANDC) with its corresponding death probability was derived. Using ANDC cut-off values of 59 and 101, COVID-19 patients were classified into three subgroups. The death probability of low risk group (ANDC < 59) was less than 5%, moderate risk group (59 ≤ ANDC ≤ 101) was 5% to 50%, and high risk group (ANDC > 101) was more than 50%, respectively.

**Conclusion:**

The prognostic nomogram exhibited good discrimination power in early identification of COVID-19 patients with high mortality risk, and ANDC score may help physicians to optimize patient stratification management.

## Introduction

Since December 2019, Coronavirus Disease 2019 (COVID-19), a newly recognized illness caused by Severe Acute Respiratory Syndrome Coronavirus 2 (SARS-CoV-2), formerly named 2019-nCoV-infected pneumonia (NCIP) broke out in Wuhan (Hubei, China) and rapidly spread throughout China and other regions of the world [1–5]. COVID-19 has caused more than 200,000 deaths around the world [5]. Although most patients with COVID-19 were mild or moderate, severe or critical cases progressed rapidly to severe pneumonia, acute respiratory distress syndrome (ARDS), coagulopathy, and septic shock, etc. [2]. Therefore, early identification of severe or critical patients is crucial to optimize patient stratification management and to potentially reduce fatality. A reliable prediction tool for mortality risk at an early stage among patients with COVID-19 would be highly valuable.

In this study, we investigated the demographics, clinical features and outcomes of patients with COVID-19, and developed a nomogram based on multiple risk factors to predict the death probability of these patients. Then, an integrated score was generated to provide a quantitative tool to early stratify COVID-19 patients and to guide the clinical management.

## Methods

### Study design

**Fig. 1:**
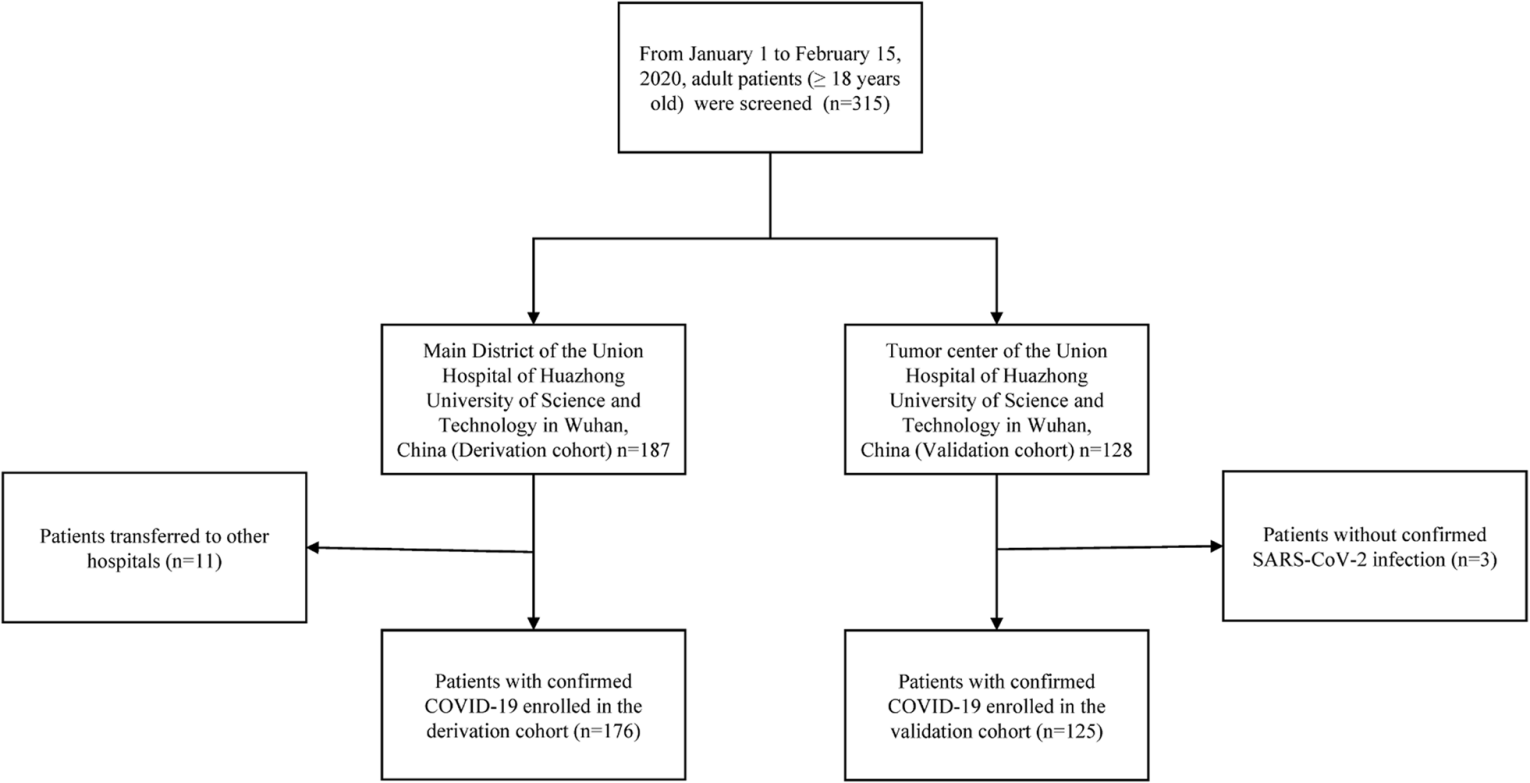
Flow chart of study participants in the derivation and validation cohort

In this retrospective study, data were collected between January 1, 2020 and February 15, 2020 from two clinical centers for COVID-19 (Main District and Tumor Center) of the Union Hospital of Huazhong University of Science and Technology (Wuhan, China). A total of 301 adult patients (≥ 18 years old) diagnosed with laboratory-confirmed COVID-19 were enrolled, 11 patients with COVID-19 in Main District transferred to other designated hospitals and 3 patients without confirmed SARS-CoV-2 infection in Tumor Center were excluded (Fig. 1). The derivation cohort consisted of 176 patients from Main District of Union Hospital (located at 1277 JieFang Avenue, Wuhan, China). The validation cohort was obtained from 125 patients admitted to Tumor Center of Union Hospital (located at 109 MaChang Road, Wuhan, China). Definite outcomes (dead or discharged) of 301 cases were followed up until March 15, 2020. All patients in this study were diagnosed according to the Guidelines of the Diagnosis and Treatment of Novel Coronavirus Pneumonia released by the China NHC [6]. This study was approved by the Ethics Committee of Tongji Medical College of Huazhong University of Science and Technology in Wuhan (2020-0058). Written informed consent was waived due to this public health emergency.

Respiratory specimens (throat swab) were collected from suspected SARS-CoV-2 infection patients for laboratory confirmation, which was performed at the local Center for Disease Control and Prevention (CDC) in Wuhan or Union Hospital (Wuhan, China). Methods for laboratory confirmation of COVID-19 had been described previously [1].

### Data collection

All patients were examined with chest computed tomography (CT) scan and additional laboratory tests on admission, including leukocytes, neutrophils, lymphocytes, alanine aminotransferase (ALT), aspartate aminotransferase (AST), lactic dehydrogenase (LDH), Creatinine, d-dimer, C-reactive protein (CRP), procalcitonin (PCT), etc. Data on epidemiological and demographic characteristics, clinical features, chronic medical histories, laboratory findings, radiological features, and clinical outcomes was obtained from the patients’ electronic medical and nursing records, and evaluated by two trained physicians (ZHW and QZ) independently. The primary outcome of this study was all-cause death.

### Statistical analysis

Discrete variables were presented as frequency and proportion, and the Fisher exact probability test was applied for comparing the difference between two groups (survivors and non-survivors). Continuous measurements were described using median and interquartile ranges (IQRs) and Mann–Whitney U tests were adopted for comparison. Random forest was applied for data imputation [7] for variables with a missing proportion of less than 10%. Those larger than 10% were excluded in the model development. A total of 24 candidate variables were involved in model development according to clinicians’ recommendations and existing literature.

The least absolute shrinkage and selection operator (LASSO) is one of the most widely used auto-variable selection techniques. In this study, LASSO was adopted for data dimensionality reduction and to enhance the model’s interpretability [8]. Five-fold cross-validation was applied to get the hyperparameter for LASSO. In order to build the most parsimonious model, we optimized LASSO by lambda within 1 SE. The predictors selected by LASSO were incorporated into a logistic regression to build the nomogram [9]. Logistic model was generally applied for a binary outcome. Since in some case, the data could not be fully interpreted by the traditional logistic model if over-dispersion, we also calculated the dispersion parameter to guarantee the model specification. Multicollinearity would lead to the difficulty of model interpretation and variance inflation factor (VIF) is a measurement to the degree of model collinearity. For this reason, we also calculated VIF to avoid the multicollinearity of the model. In internal validation, we applied Hosmer–Lemeshow test for the goodness-of-fit of the model and drew the 1000-times-bootstrapping resampling calibration plot [9] for internal calibration. Internal discrimination was assessed by the median of the area under the curve (AUC) for receiver operating characteristic (ROC) of the logistic model through 1000-times-repeated bootstrapping resampling. Similarly, we redrew a calibration plot and estimate AUC for retesting model performance in external-validation cohort to avoid overfitting. Decision curve analysis takes both discrimination and calibration into consideration. In this analysis, we used it [10] for comparison of the performance between single-predictor models and the full model. We further classified the patients into three subgroups based on the total points derived from the predictive nomogram. The Fisher’ exact test was applied to compare the actual fatality rates among the three subgroups.

In this study, the two-tailed test was conducted apart from the one-tailed Hosmer–Lemeshow test and significance thresholds were set at 0.05 for the two-tailed test and 0.025 for the one-tailed. In addition, we applied Bonferroni correction to adjust significance thresholds for multiple comparisons. All statistical analyses were performed using the software R version 3.6.2 (https://www.r-project.org/). The following packages were used: “missForest”, “glmnet”, “rms”, “rmda”, “pROC” and “ggplot2″.

## Results

### Characteristics of COVID-19 patients

A total of 301 COVID-19 patients were enrolled from two clinical centers of Union Hospital (Wuhan, China) (Fig. 1). Among 301 cases with laboratory-confirmed COVID-19, the case fatality rates (CFR) of the derivation and validation cohorts were 11.9% (21/176) and 8.8% (11/125), respectively (p = 0.451). The proportion of sex and diabetes were comparable between the two cohorts (p > 0.05, Table 1), while the proportion of age, hypertension, coronary heart disease, days from illness onset to admission and days from illness onset to discharge or death were different between the two cohorts (p < 0.05, Table 1).

**Table 1:** Characteristics between the derivation cohort and validation cohort

	Derivation cohort	Validation cohort	*P* value	Overall
	(N = 176)	(N = 125)		(N = 301)
Outcome			0.451	
Non-survival, n (%)	21 (11.9)	11 (8.8)		32 (10.6)
Survival, n (%)	155 (88.1)	114 (91.2)		269 (89.4)
Gender			0.411	
Female, n (%)	103 (58.5)	67 (53.6)		170 (56.5)
Male, n (%)	73 (41.5)	58 (46.4)		131 (43.5)
Age, years	47.0 (33.0–62.0)	68.0 (62.0–73.0)	< 0.001	61.0 (41.0–69.0)
Comorbidity, n (%)	37 (21.0)	60 (48.0)	< 0.001	97 (32.2)
Diabetes, n (%)	21 (11.9)	18 (14.4)	0.602	39 (13.0)
Hypertension, n (%)	24 (13.6)	45 (36.0)	< 0.001	69 (22.9)
Coronary heart disease, n (%)	9 (5.1)	17 (13.6)	0.012	26 (8.6)
Illness onset to admission, days	7.0 (5.0–10.0)	12.0 (8.0–20.0)	< 0.001	9.0 (6.0–14.0)
Illness onset to discharge or death, days	21.0 (16.0–26.0)	38.0 (33.0–46.0)	< 0.001	27.0 (19.0–38.0)

The details for characteristics of survivors and non-survivors of the derivation cohort were summarized in Table 2. The median age of the participants in the derivation cohort was 47.0 (33.0–62.0) years and more than half of them were female (58.5%). Non-survivors were older than survivors (70.0 vs 43.0 years, p < 0.001). The proportions of diabetes, hypertension and coronary heart disease were significantly different between the two groups (Table 2). In particular, no significant difference was found in the median time from illness onset to hospital admission between survivors and non-survivors (p = 0.391). Compared with survivors, non-survivors had increased white blood cells (7.2 vs 4.4 × 10^9^/L, p < 0.001), higher neutrophil counts (6.3 vs 2.8 × 10^9^/L, p < 0.001), lower lymphocyte counts (0.66 vs 1.06 × 10^9^/L, p < 0.001), higher CRP levels (83.15 vs 13.70 mg/L, p < 0.001), higher d-dimer levels (1.85 vs 0.39 mg/L, p < 0.001), and higher lactate dehydrogenase levels (451.0 vs 227.0 U/L, p < 0.001).

**Table 2:** The characteristics of patients with COVID-19 in derivation cohort

	Survivors	Non-survivors	All patients	*p* value^a^
	(n = 155)	(n = 21)	(n = 176)	
Gender				0.004
Female, n (%)	97 (62.6)	6 (28.6)	103 (58.5)	
Male, n (%)	58 (37.4)	15 (71.4)	73 (41.5)	
Age, median (IQR), years	43.0 (32.0–59.0)	70.0 (65.0–76.0)	47.0 (33.0–62.0)	< 0.001
Comorbidity, n (%)	25 (16.1)	12 (57.1)	37 (21.0)	< 0.001
Diabetes, n (%)	14 (9.0)	7 (33.3)	21 (11.9)	0.005
Hypertension, n (%)	16 (10.3)	8 (38.1)	24 (13.6)	0.002
Coronary heart disease, n (%)	3 (1.9)	6 (28.6)	9 (5.1)	< 0.001
Illness onset to admission, median (IQR), days	7.0 (5.0–10.7)	7.0 (5.0–8.0)	7.0 (5.0–10.0)	0.391
Illness onset to discharge or death, median (IQR), days	21.0 (17.0–27.0)	17.0 (14.0–20.0)	21.0 (16.0–26.0)	<0.001
Complication, n (%)	8 (5.2)	18 (85.7)	26 (14.7)	< 0.001
ARDS, n (%)	4 (2.6)	18 (85.7)	22 (12.5)	< 0.001
Acute renal injury, n (%)	0	1 (4.8)	1 (0.6)	0.119
Acute cardiac injury, n (%)	4 (2.6)	2 (9.5)	6 (3.4)	0.152
Septic shock, n (%)	0	5 (23.8)	5 (2.8)	< 0.001
White blood cells, median (IQR), ×10^9^/L	4.4 (3.2–5.5)	7.2 (6.6–9.9)	4.6 (3.4–6.1)	< 0.001
Hemoglobin, median (IQR), g/L	126.0 (116.3–136.0)	119.0 (114.0–133.0)	125.0 (116.0–136.0)	0.275
Platelet, median (IQR), ×10^6^/L	189.0 (140.0–232.0)	142.0 (128.0–203.0)	182.0 (137.5–232.0)	0.039
Neutrophils, median (IQR), ×10^9^/L	2.78 (1.90–3.71)	6.30 (5.07–8.47)	2.90 (2.06–4.41)	< 0.001
Neutrophils%, median (IQR)	62.0 (54.3–75.1)	86.40 (83.10–91.00)	63.35 (55.25–77.50)	< 0.001
Lymphocytes, median (IQR), ×10^9^/L	1.06 (0.81–1.40)	0.66 (0.55–0.80)	0.99 (0.73–1.39)	< 0.001
Lymphocytes%, median (IQR)	27.6 (16.7–35.3)	8.5 (5.3–12.5)	25.4 (14.5–34.0)	< 0.001
NLR, median (IQR)	2.3 (1.5–4.3)	10.6 (6.9–17.3)	2.6 (1.6–5.2)	< 0.001
Total bilirubin, median (IQR), μmol/L	9.1 (7.4–11.8)	16.3 (13.9–19.3)	9.5 (7.6–12.8)	< 0.001
Direct bilirubin, median (IQR), μmol/L	3.4 (2.5–4.6)	7.1 (5.9–10.7)	3.5 (2.6–5.2)	< 0.001
Alanine aminotransferase, median (IQR), U/L	23.0 (16.0–35.0)	30.0 (23.0–54.0)	23.0 (17.0–39.0)	0.015
Aspartate aminotransferase, median (IQR), U/L	25.0 (20.0–36.0)	41.00 (31.0–55.0)	26.50 (20.0–41.0)	<0.001
Lactate dehydrogenase, median (IQR), U/L	227.0 (181.0–323.8)	451.0 (358.0–516.0)	240.0 (185.5–350.0)	< 0.001
Creatine kinase, median (IQR), U/L	57.0 (42.0–95.5)	129.0 (66.5–177.5)	59.50 (42.3–105.8)	0.006
Blood urea nitrogen, median (IQR), mmol/L	3.6 (2.9–4.6)	5.4 (4.5–8.1)	3.8 (3.0–5.0)	< 0.001
Creatinine, median (IQR), μmol/L	65.8 (57.4–75.9)	79.1 (62.5–86.7)	66.3 (57.4–79.2)	0.069
Serum potassium, median (IQR), mmol/L	3.9 (3.6–4.2)	3.5 (3.2–4.1)	3.9 (3.6–4.2)	0.033
Serum sodium, median (IQR), mmol/L	139.8 (137.6–141.4)	136.1 (133.9–139.5)	139.5 (137.3–141.3)	0.001
d-Dimer, median (IQR), mg/L	0.39 (0.22–0.80)	1.85 (1.52–5.95)	0.44 (0.22–1.06)	< 0.001
Prothrombin time, median (IQR), s	13. (12.7–13.5)	14.1 (13.4–14.7)	13.1 (12.8–13.7)	< 0.001
Thrombin time, median (IQR), s	17.3 (16.5–18.5)	17.5 (15.2–18.4)	17.3 (16.4–18.5)	0.519
APTT, median (IQR), s	38.3 (36.4–41.7)	38.75 (35.10–41.50)	38.35 (36.2–41.7)	0.877
INR, median (IQR),	1.0 (1.0–1.1)	1.1 (1.0–1.2)	1.0 (1.0–1.1)	< 0.001
Fibrinogen, median (IQR), g/L	4.3 (3.7–5.4)	5.2 (3.8–6.2)	4.4 (3.7–5.5)	0.231
C-reactive protein, median (IQR), mg/L	13.7 (3.8–38.5)	83.2 (55.5–149.6)	16.7 (4.7–52.9)	< 0.001
Procalcitonin, median (IQR), μg/L	0.13 (0.13–0.13)	0.15 (0.13–0.56)	0.13 (0.13–0.13)	< 0.001

*COVID*-*19* coronavirus disease 2019, *IQR* interquartile range, *ARDS* acute respiratory distress syndrome, *NLR* neutrophils-to-lymphocytes ratio, *APTT* activated partial thromboplastin time, *INR* international normalized ratio

^a^*p* values indicate differences between survivors and non-survivors. *p *< 0.05 was considered statistically significant

On admission, all patients in the derivation cohort had pneumonia which was diagnosed by chest CT scan and 161 (91.5%) patients’ CT images showed bilateral lung impairment. All patients received antiviral treatment, such as ribavirin, arbidol hydrochloride, lopinavir and ritonavir or interferon-α2b (nebulization inhalation). Other symptomatic and supportive treatments were performed according to the Guidelines of the Diagnosis and Treatment of Novel Coronavirus Pneumonia published by the China NHC [6]. Acute respiratory distress syndrome, septic shock, acute cardiac injury, and acute renal injury were the common complications (Table 2).

### Development of the nomogram

**Fig. 2:**
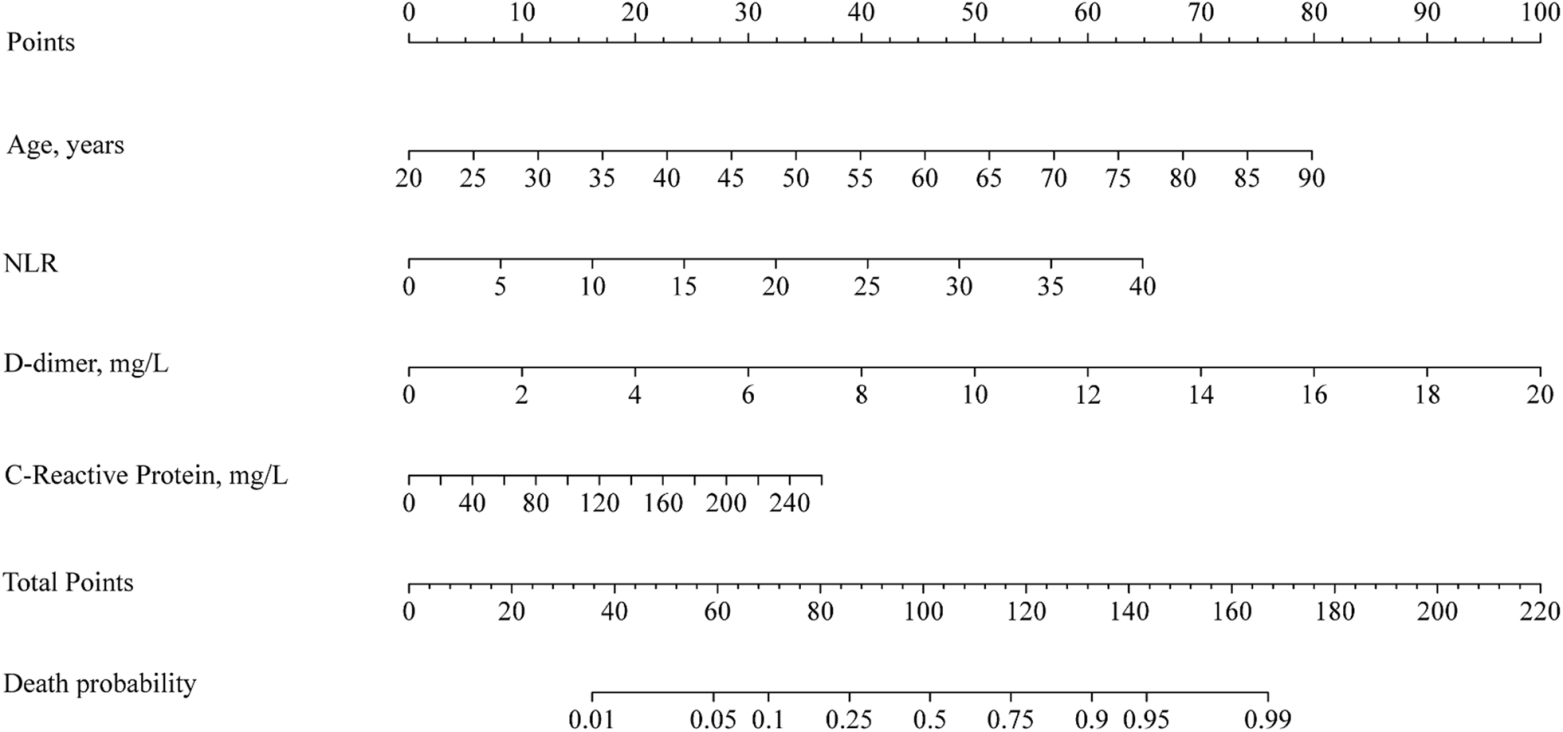
Nomogram to predict the death probability of patients with COVID-19. The nomogram was constructed based on the following variables: age, NLR, D-dimer and CRP. Locate the values of a patient’s age, NLR, D-dimer, and CRP and draw four vertical lines for each of the four predictors to reach the “Points” axis, respectively. The intersections between the vertical lines and the “Points” axis are the corresponding score for the predictors. The summation of the scores from four predictors (named ANDC) could be converted to death probability of patients with COVID-19 by drawing another vertical line from the “Total points” axis to the “Death probability” axis. COVID-19, coronavirus disease 2019; NLR, neutrophils-to-lymphocytes ratio; CRP, C-reactive protein

The nomogram (Fig. 2), containing four variables: age, NLR, d-dimer, and CRP, is a regression model visualization for evaluating death probability. Here is the instruction of the nomogram: locate the values of a patient’s age, NLR, d-dimer, and CRP and draw four vertical lines for each of the four predictors to reach the “Points” axis, respectively. The intersections between the vertical lines and the “Points” axis are the corresponding score for the predictors. The summation of the scores from four predictors (named ANDC) could be converted to death probability in the same way. In that, the clinicians could easily predict the death probability and identify the high-risk patient. In general, the higher value of ANDC, the greater probability of death (Fig. 2).

Alternatively, the ANDC score also could be calculated by using the following formula: ${\text{Total points }}\left( {\text{ANDC}} \right) = \left( {1.14 \times {\text{age}} - 20} \right)\left( {\text{years}} \right) + 1.63 \times {\text{NLR}} + 5.00 \times {\text{D}} - {\text{dimer}}\left( {{\text{mg}}/{\text{L}}} \right) + 0.14 \times {\text{CRP}}\left( {{\text{mg}}/{\text{L}}} \right)$. We provided a list about the specific ANDC score and corresponding death probability at Additional file 1: Table S1. In particular, an ANDC of 59 and 101 corresponded to the 5% and 50% cutoffs of death probability, respectively. We suggested that 59 and 101 could be used as cutoff values to stratify COVID-19 patients into three groups. The death probability of low risk group (ANDC < 59) was less than 5%, moderate risk group (59 ≤ ANDC ≤ 101) was between 5% and 50%, and high-risk group (ANDC > 101) was more than 50%, respectively.

Furthermore, we compared the actual death proportion with the predicted death probability in the three classified subgroups according to the ANDC score. As shown in Additional file 2: Table S2, the proportions of death were 0.9% (1/110) for low risk group, 18.0% (9/50) for moderate risk group and 68.8% (11/16) for high-risk group. The actual fatality rates were significant different (p < 0.001) among the three subgroups.

### Performance of the nomogram

**Fig. 3:**
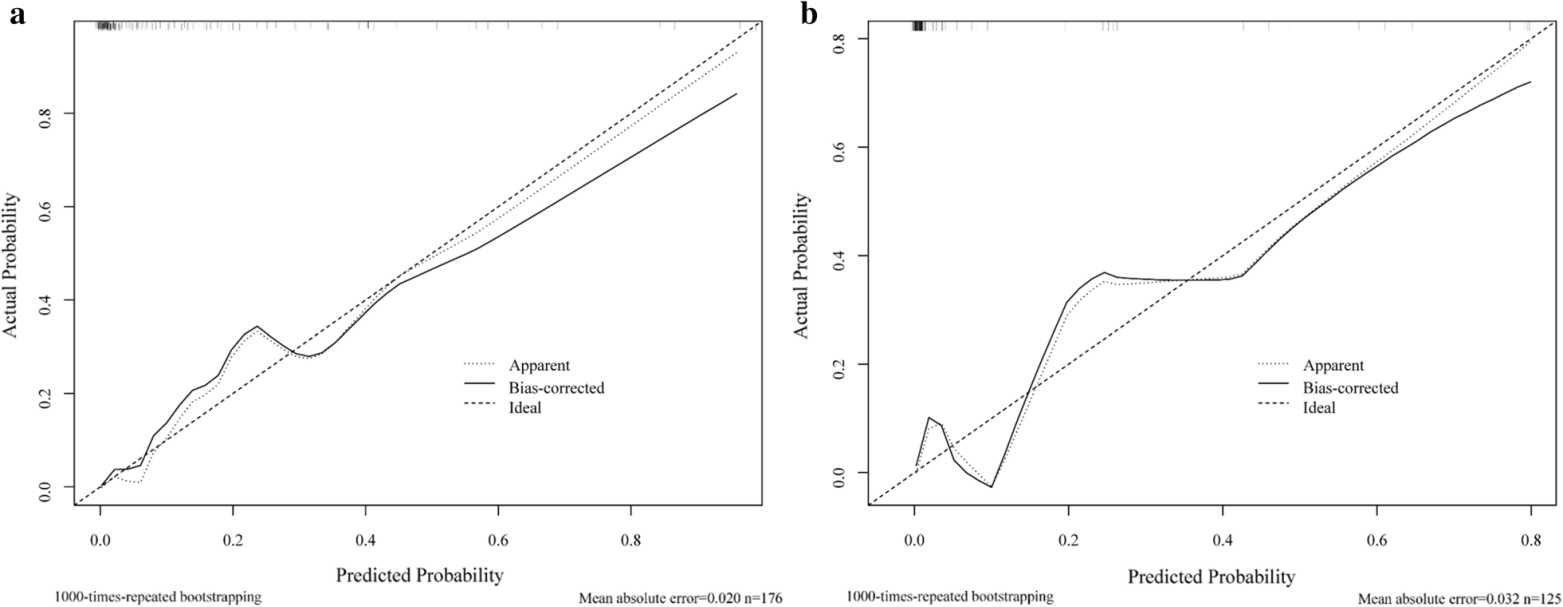
Calibration plot comparing predicted and actual death probability of patients with COVID-19. These two figures show actual against predicted death probability of patients with COVID-19. **a** represents the internal validation. **b** Represents the external validation. Dotted curve represents the apparent curve without bootstrapping correction. The solid curve represents the 1000-times repeated bootstrapping-correction curve. The dashed curve represents the ideal fit. COVID-19, coronavirus disease 2019

The dispersion parameter was 0.382 less than 1 and the maximum of VIF of predictors in the full model is less than 1.25, which showed the non-existence of over-dispersion and multicollinearity. *p* value of the Hosmer–Lemeshow test was 0.751 greater than 0.025, which demonstrated consistency between actual probability and observed probability of the outcome. In addition, according to Fig. 3, the biased-corrected curve in calibration plot graphed closely toward the diagonal line, representing the consistent conclusion under bootstrapping correction conditions.

**Fig. 4:**
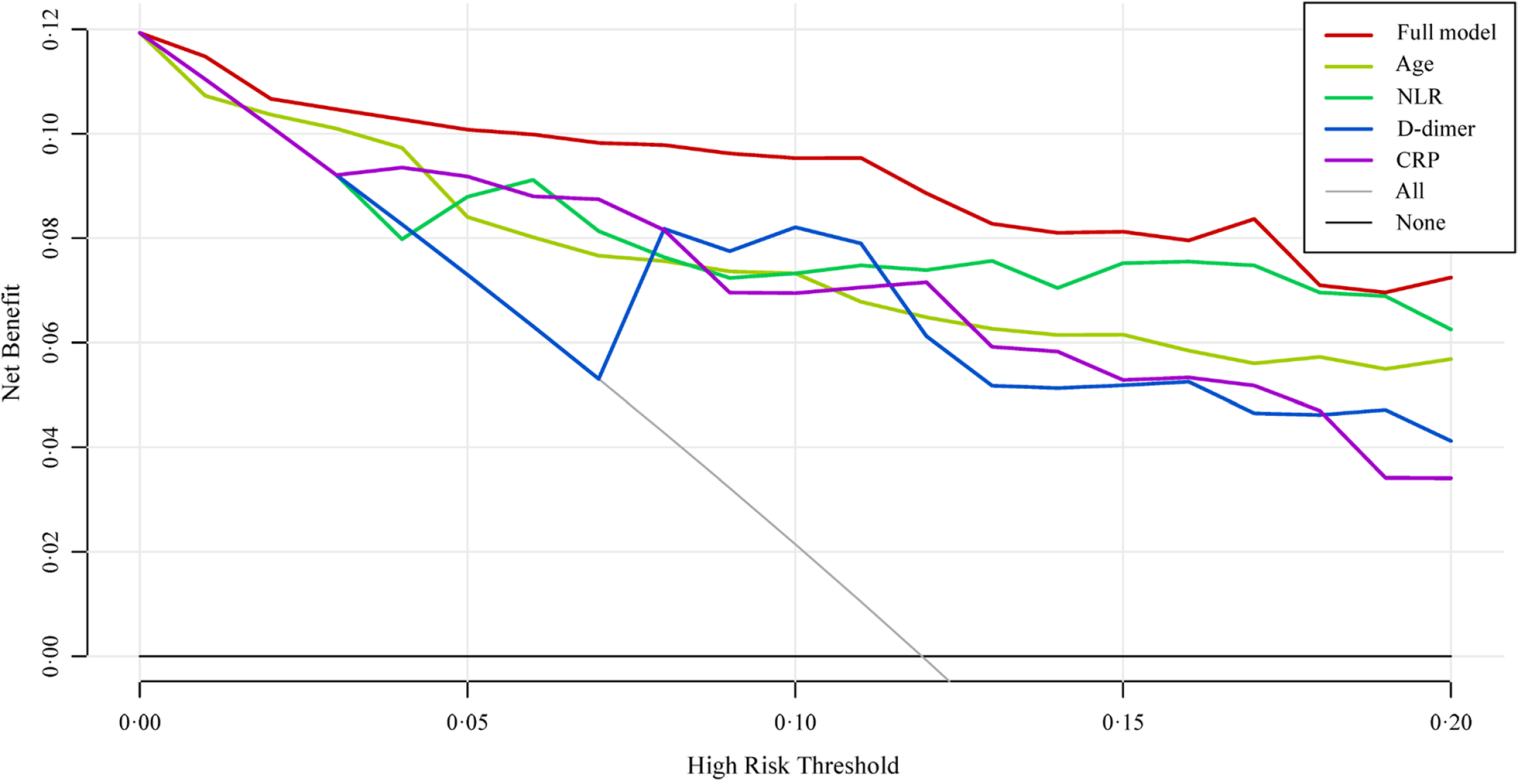
Decision curves analysis comparing different models to predict the death probability of patients with COVID-19. The net benefit balances the mortality risk and potential harm from unnecessary over-intervention for patients with COVID-19. Full model incorporates the following predictors: age, NLR, D-dimer and CRP. *COVID-19* coronavirus disease 2019, *NLR* neutrophils-to-lymphocytes ratio, *CRP* C-reactive protein

Our model’s discrimination statistics AUC was 0.921 (95% CI 0.835–0.968) under bootstrapping correction. Based on Fig. 4, the net benefit of every single predictor model was positive, indicating every predictor contributed to the prediction of outcomes. In particular, the full model demonstrated the best performance and hence it was necessary to combine four predictors in the model.

### External validation

It is essential to evaluate the model performance by the cohort which is not used for developing prediction model to avoid overfitting. In this study, we performed a series of external validation analysis. The patients in the validation cohorts were divided into three classified subgroups according to the ANDC score. As shown in Additional file 3: Table S3, the proportions of death were 0.0% (0/35) for low risk group, 1.4% (1/71) for moderate risk group and 52.6% (10/19) for high risk group. The actual fatality rates were significant different (p < 0.001) among the three subgroups. In consistent with the derivation cohort, the model still performed well in AUC of 0.975 (95% CI 0.947–1.000) and calibration plot was indicative of the reliable model even under the context of an external dataset (Fig. 3).

## Discussion

In this study, we investigated the correlation between the outcomes of COVID-19 patients and multiple factors. Several indicators were identified by LASSO regression analysis as predictors for COVID-19 patients with a poor outcome including age, NLR, D-dimer and CRP. Then, a visualized nomogram was established based on these four variables, and ANDC scores could be easily obtained for early stratifying COVID-19 patients and improving the clinical management for the disease Additional files 4, 5: Figures S1, S2.

The majority of patients with COVID-19 were mild [4]. Most mild cases were recommended oral medications and self-isolation at home at the initial stage of this outbreak, which may result in the relatively higher CFR among inpatients in our derivation cohort. In addition, Tumor Center of Union Hospital was the designated clinical center for patients with COVID-19 since February, 2020. Therefore, severe or critical COVID-19 patients were transferred to there from other hospitals, which also possibly contributed to the higher CFR among hospitalized patients in the validation cohort in our study than previously reported [3, 4].

In accordance with previous studies on severe acute respiratory syndrome (SARS) [11], Middle East respiratory syndrome (MERS) [12] and COVID-19 [13], older age was also identified as a predictor for poor prognosis of patients with COVID-19 in our study. It is hypothesized that immunosenescence and/or underlying comorbidities might deem geriatric patients more vulnerable to developing severe COVID-19 illness.

Recently, Liu et al. [14] proposed that increased NLR was a risk factor for the early identification of severe COVID-19 illness. In this study, we found that higher NLR was associated with higher mortality. In patients with community-acquired pneumonia, an extensive activation of the immune system and/or immune dysfunction can lead to alterations of the ratio between serum neutrophil and lymphocyte levels [15]. Moreover, when there are immunosuppression and apoptosis of lymphocytes induced by various anti-inflammatory cytokines, neutrophils, especially immature neutrophils are recruited from the bone marrow into the cycle [16], resulting in an increased NLR. Research performed by Lu et al. [17] suggested that CRP tested on admission can predict confirmed or suspected COVID-19 related short-term mortality. CRP is synthesized by hepatocytes in response to cytokines which are derived from leukocytes stimulated by infection, inflammation, or tissue damage. Recently, CRP is widely used clinically to evaluate disease progression, and it served as an indicator for predicting bacterial infections in patients [18–20]. In our study, increased CRP levels measured at admission of patients with COVID-19 was associated with increased mortality risk. This suggested that a severe inflammation or potentially a secondary infection has developed in these patients, and empirical antibiotic treatment might be necessary.

A previous study had suggested that the dysregulation of the urokinase, coagulation and fibrinolysin pathways during SARS-CoV and influenza virus infections results in more severe lung pathologies, by disturbing the balance between host coagulation and fibrinolysin pathways [21]. d-dimer is often regarded as an indicator for fibrinolytic system activity. Once inflammation has occurred, the alveolar hemostatic balance is shifted to prominently exhibit procoagulant activity, resulting high d-dimer levels [22]. Furthermore, inflammatory cytokines can also activate coagulation cascade and inhibit fibrinolysis in patients with severe sepsis [23]. On the other hand, d-dimer has been demonstrated as a major indicator for diagnosing pulmonary embolism (PE) [24], which also affects prognosis. In the current study, 21 fatal cases demonstrated elevated D-dimer levels on admission in the derivation cohort, which might indicate that a therapeutic approach targeting coagulopathy-related signaling pathway should be considered at that time.

Based on the above analyses, these four mortality predictors in our nomogram were associated with inflammation, immunity and coagulation function, which might contribute to the pathogenesis of COVID-19. We speculated that the inflammatory response to SARS-CoV-2 infection may be the core in the pathogenesis of COVID-19, and the dysregulation of the immune and/or coagulation system will result in worse disease outcomes, such as ARDS, coagulopathy, and septic shock, etc. In our study, non-survivors had low levels lymphocytes and higher levels of neutrophils, D-dimer and CRP than those of survivors. An early intervention based on comprehensive consideration of inflammatory response, immune dysfunction and coagulopathy might contribute to make a reasonable and individualized therapeutic strategy for COVID-19 patients with high mortality risk.

Lately, Chen et al. proposed that older age, dyspnea, coronary heart disease, cerebrovascular disease, elevated PCT and AST are independent risk factors associated with fatal outcome and developed a nomogram to predict the survival of patient with COVID-19 in China but without external validation. In our study, the aforementioned four predictors (age, NLR, D-dimer and CRP) obtained on admission were selected by the LASSO analysis to construct a predictive nomogram, which exhibited good discrimination and calibration in the individualized prediction for the death probability of COVID-19 patients. Furthermore, our nomogram was validated by an external heterogeneous cohort and it appeared to be useful in different clinical settings. The application of our nomogram in the derivation and validation cohort showed good differentiation with AUC values of 0.912 and 0.975, which were higher than Chen’s nomogram (AUC = 0.849). Moreover, the ANDC score derived from the nomogram provided a quantitative tool for the early identification of patients with high mortality risk on admission and for guiding clinical managements. Patients with COVID-19 was classified by the ANDC score obtained on admission into three risk groups with varied mortality risk. Cases in the low risk group should be isolated and treated in “Mobile Cabin Hospitals” [17]. Patients with moderate risk should be admitted to a designated hospital for comprehensive treatments in an isolation ward. Patients with high risk should be intensive surveillance and should be transferred to ICU for aggressive treatment and critical supportive care if necessary.

There were several limitations in our study. Firstly, this is a retrospective study and hence the model needs to be validated by multicenter prospective studies. Secondly, patients with elevated D-dimer levels on admission may indicate that the underlying high risk PE status possibly occurred. Owing to the retrospective study design, CT angiography used to diagnose PE was not performed in all COVID-19 patients.

## Conclusions

In summary, based on multiple risk factors (age, NLR, D-dimer and CRP), our nomogram for predicting the prognosis of patients with COVID-19 showed good discrimination and calibration. The application of ANDC would help clinicians make a prompt and reasonable decision to optimize patient stratification management and to potentially reduce fatality. However, this quantitative tool needs to be validated by further large-scale prospective studies.

## Supplementary information

**Additional file 1: Table S1.** Total points in nomogram and corresponding death probability of patients with COVID-19.

**Additional file 2: Table S2.** The association between different risk groups and actual outcome in the derivation cohort.

**Additional file 3: Table S3.** The association between different risk groups and actual outcome in the validation cohort.

**Additional file 4: Figure S1.** Five-fold cross-validation to select the Lambda.1SE for LASSO based on binominal deviance.

**Additional file 5: Figure S2.** LASSO coefficient profiles of the 24 candidate predictors.

## Data Availability

All data analyzed during the current study are included in this article.

## Abbreviations

COVID-19: Coronavirus Disease 2019
SARS-CoV-2: Severe Acute Respiratory Syndrome Coronavirus 2
NCIP: 2019-nCoV-infected pneumonia
ARDS: Acute respiratory distress syndrome
CDC: Center for Disease Control and Prevention
LASSO: Least absolute shrinkage and selection operator
CFR: Case fatality rates
NLR: Neutrophil-to-lymphocyte ratio
SARS: Severe acute respiratory syndrome
MERS: Middle East respiratory syndrome
