# Supplementary material for: ANDC: an early warning score to predict mortality risk for patients with Coronavirus Disease 2019
Source: J Transl Med. 2020 Aug 31;18:328. doi: 10.1186/s12967-020-02505-7 (PMC7457219; doi:10.1186/s12967-020-02505-7)
Supplement: Supplementary file 1 — Additional file 1: Table S1. Total points in nomogram and corresponding death probability of patients with COVID-19. [file 12967_2020_2505_MOESM1_ESM.docx]

| **Additional Table S1 Total points in nomogram and corresponding death probability of patients with COVID-19** | |
| --- | --- |
| **Total Points** | **Death probability** |
| 36 | 0.01 |
| 59 | 0.05 |
| 70 | 0.10 |
| 86 | 0.25 |
| 101 | 0.50 |
| 117 | 0.75 |
| 133 | 0.90 |
| 143 | 0.95 |
| 167 | 0.99 |

COVID-19, coronavirus disease 2019.

Total points$=\left( 1.14\times age-20 \right)\left( \mathrm{years} \right)+1.63\times NLR+5.00\times D-dimer(mg/L)+0.14\times CRP(mg/L)$

Linear prediction $=-8.680888+ 0.079714\times age \left( \mathrm{years} \right)+0.113387\times NLR + 0.349651\times D-dimer (mg/L) + 0.009809\times CRP (mg/L)$

Death probability = 1/ (1+exp (-Linear Prediction)).
