# Supplementary material for: ANDC: an early warning score to predict mortality risk for patients with Coronavirus Disease 2019
Source: J Transl Med. 2020 Aug 31;18:328. doi: 10.1186/s12967-020-02505-7 (PMC7457219; doi:10.1186/s12967-020-02505-7)
Supplement: Supplementary file 2 — Additional file 2: Table S2. The association between different risk groups and actual outcome in the derivation cohort. [file 12967_2020_2505_MOESM2_ESM.docx]

| **Additional Table S2 The association between different risk groups and actual outcome in the derivation cohort^a^** | | | |
| --- | --- | --- | --- |
| **Risk category** | **Outcome** | | **Overall** |
|  | **Alive** | **Death** |  |
| Low risk | 109 (99.1) | 1 (0.9) | 110 (100.0) |
| Moderate risk | 41 (82.0) | 9 (18.0) | 50 (100.0) |
| High risk | 5 (31.2) | 11 (68.8) | 16 (100.0) |
| Overall | 155 (88.1) | 21 (11.9) | 176 (100.0) |
| a: Values are numbers (percentages) unless stated otherwise. Fisher exact probability test was applied. Bonferroni correction was used for pairwise comparisons and significant threshold was corrected as 0.05/3=0.017. Denoting: P-value of A vs B represents comparing the proportion of outcome between A and B.  P-value among three group is less than 0.001  P-value of Low-risk group vs Moderate-risk group is less than 0.001.  P-value of Low-risk group vs High-risk group is less than 0.001.  P-value of Moderate-risk group vs High-risk group is less than 0.001. | | | |
