# Supplementary material for: ANDC: an early warning score to predict mortality risk for patients with Coronavirus Disease 2019
Source: J Transl Med. 2020 Aug 31;18:328. doi: 10.1186/s12967-020-02505-7 (PMC7457219; doi:10.1186/s12967-020-02505-7)
Supplement: Supplementary file 3 — Additional file 3: Table S3. The association between different risk groups and actual outcome in the validation cohort. [file 12967_2020_2505_MOESM3_ESM.docx]

| **Additional Table S3 The association between different risk groups and actual outcome in the validation cohort^a^** | | | |
| --- | --- | --- | --- |
| **Risk category** | **Outcome** | | **Overall** |
|  | **Alive** | **Death** |  |
| Low risk | 35 (100.0) | 0 | 35 (100.0) |
| Moderate risk | 70 (98.6) | 1 (1.4) | 71 (100.0) |
| High risk | 9 (47.4) | 10 (52.6) | 19 (100.0) |
| Overall | 114 (91.2) | 11 (8.8) | 125 (100.0) |
| a: Values are numbers (percentages) unless stated otherwise. Fisher exact probability test was applied. Bonferroni correction was used for pairwise comparisons and significant threshold was corrected as 0.05/3=0.017. Denoting: P-value of A vs B represents comparing the proportion of outcome between A and B. P-value among three group is less than 0.001 P-value of Low-risk group vs Moderate-risk group is 1. P-value of Low-risk group vs High-risk group is less than 0.001. P-value of Moderate-risk group vs High-risk group is less than 0.001. | | | |
