# Supplementary material for: ANDC: an early warning score to predict mortality risk for patients with Coronavirus Disease 2019
Source: J Transl Med. 2020 Aug 31;18:328. doi: 10.1186/s12967-020-02505-7 (PMC7457219; doi:10.1186/s12967-020-02505-7)
Supplement: Supplementary file 4 — Additional file 4: Figure S1. Five-fold cross-validation to select the Lambda.1SE for LASSO based on binominal deviance. [file 12967_2020_2505_MOESM4_ESM.docx]

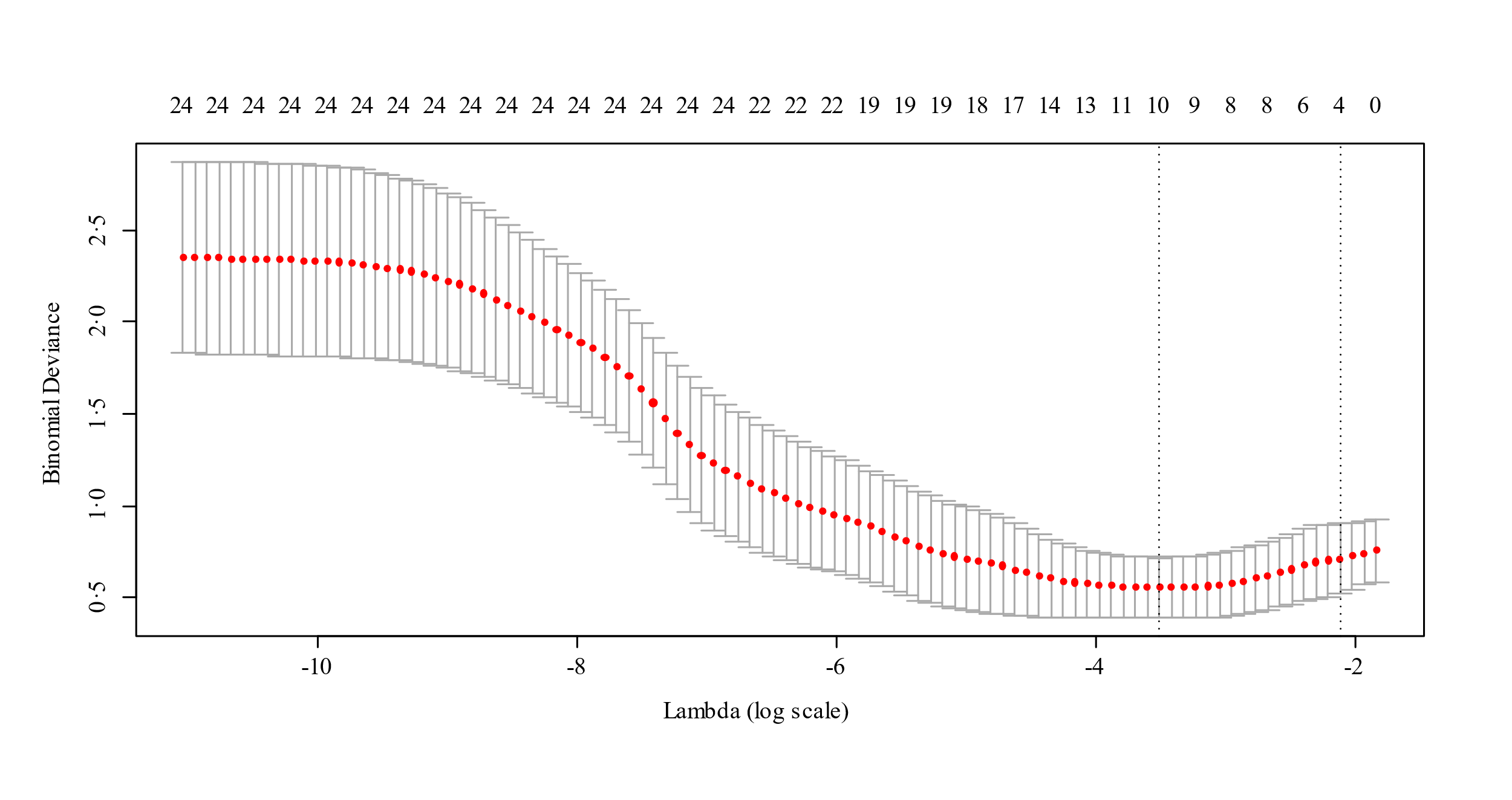


**Additional Fig. S1 Five-fold cross-validation to select the Lambda.1SE for LASSO based on binominal deviance.** From left to right, the first dotted line represents the location of the minimal of lambda and the second represents the Lambda.1SE. Based on “one-standard-error” rule, Lambda.1SE of 0.1206015 (-2.115264 in log scale) was selected as the tune parameter for LASSO through 5-fold cross-validation, which resulted in 4 predictors with nonzero coefficients including age, neutrophils-to-lymphocytes ratio, D-dimer and C-reactive protein. Lambda.1SE, lambda within 1 standard error; LASSO, least absolute shrinkage and selection operator.
