# Supplementary material for: ANDC: an early warning score to predict mortality risk for patients with Coronavirus Disease 2019
Source: J Transl Med. 2020 Aug 31;18:328. doi: 10.1186/s12967-020-02505-7 (PMC7457219; doi:10.1186/s12967-020-02505-7)
Supplement: Supplementary file 5 — Additional file 5: Figure S2. LASSO coefficient profiles of the 24 candidate predictors. [file 12967_2020_2505_MOESM5_ESM.docx]

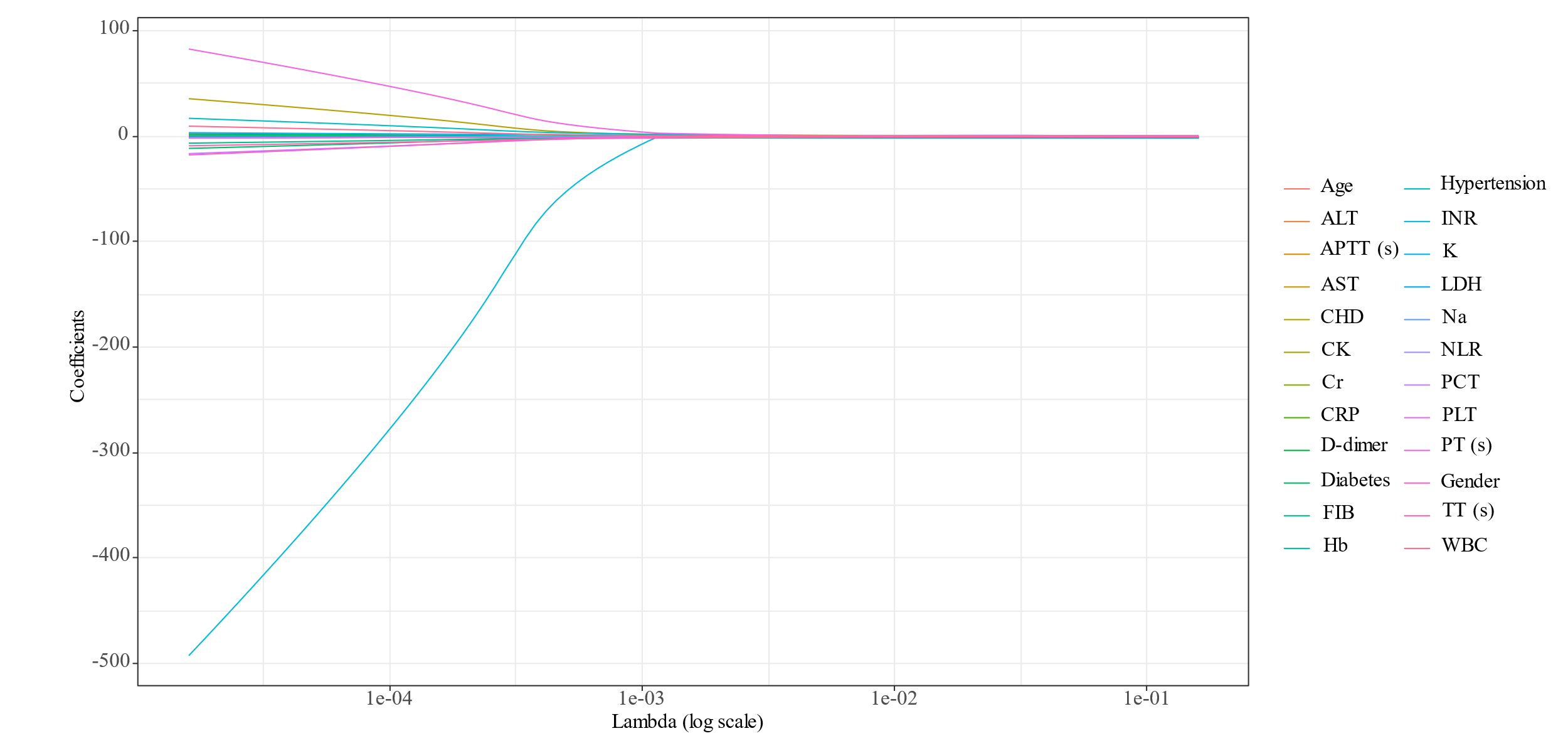


**Additional Fig. S2 LASSO coefﬁcient proﬁles of the 24 candidate predictors.** ALT, alanine aminotransferase; INR, international normalized ratio; APTT, activated partial thromboplastin time; K, serum potassium; AST, aspartate aminotransferase; LDH, lactate dehydrogenase; CHD, coronary heart disease; Na, serum sodium; CK, creatine kinase; NLR, neutrophils-to-lymphocytes ratio; Cr, creatinine; PCT, procalcitonin; CRP, C-reactive protein; PLT, platelet; PT, prothrombin time; FIB, fibrinogen; TT, thrombin time; Hb, hemoglobin; WBC, white blood cells; LASSO, Least absolute shrinkage and selection operator.
